# Serum Concentrations of Antibodies Against Vaccine Toxoids in Children Exposed Perinatally to Immunotoxicants

**DOI:** 10.1289/ehp.1001975

**Published:** 2010-06-20

**Authors:** Carsten Heilmann, Esben Budtz-Jørgensen, Flemming Nielsen, Birger Heinzow, Pál Weihe, Philippe Grandjean

**Affiliations:** 1 Paediatric Clinic II, Copenhagen University Hospital, Rigshospitalet, Copenhagen, Denmark; 2 Department of Biostatistics, University of Copenhagen, Copenhagen Denmark; 3 Institute of Public Health, University of Southern Denmark, Odense, Denmark; 4 State Social Services Agency, Department for Healthcare, Kiel, Germany; 5 Department of Occupational Medicine and Public Health, Tórshavn, Faroe Islands; 6 Department of Environmental Health, Harvard School of Public Health, Boston, Massachusetts, USA

**Keywords:** developmental toxicity, environmental exposure, immunotoxicity, methylmercury, polychlorinated biphenyls

## Abstract

**Background:**

Polychlorinated biphenyls (PCBs) may cause immunotoxic effects, but the detailed dose–response relationship and possible vulnerable time windows of exposure are uncertain. In this study we applied serum concentrations of specific antibodies against childhood vaccines as sentinels of immunotoxicity.

**Objectives:**

The main objective was to assess the possible dependence of antibody concentrations against diphtheria and tetanus toxoids in children with regard to prenatal and postnatal PCB exposures.

**Methods:**

From a cohort of 656 singleton births formed in the Faroe Islands during 1999–2001, children were invited for examination with assessment of serum antibody concentrations at 5 years (before and after a booster vaccination) and at 7 years of age. Total PCB concentrations were determined in serum from ages 5 and 7 years, and data were also available on PCB concentrations in maternal pregnancy serum, maternal milk, and, for a subgroup, the child’s serum at 18 months of age.

**Results:**

A total of 587 children participated in the examinations at ages 5 and/or 7 years. At age 5 years, before the booster vaccination, the antidiphtheria antibody concentration was inversely associated with PCB concentrations in milk and 18-month serum. Results obtained 2 years later showed an inverse association of concentrations of antibodies against both toxoids with PCB concentrations at 18 months of age. The strongest associations suggested a decrease in the antibody concentration by about 20% for each doubling in PCB exposure. At age 5 years, the odds of an antidiphtheria antibody concentration below a clinically protective level of 0.1 IU/L increased by about 30% for a doubling in PCB in milk and 18-month serum.

**Conclusions:**

Developmental PCB exposure is associated with immunotoxic effects on serum concentrations of specific antibodies against diphtheria and tetanus vaccinations. The immune system development during the first years of life appears to be particularly vulnerable to this exposure.

Polychlorinated biphenyls (PCBs) have been commercially produced since the 1920s and were identified as environmental pollutants in 1966 (Beyer and Biziuk 2009). They have been recently suspected of causing immunotoxic effects because of their similarity to dioxins (Larsen 2006). As a model immunotoxicant, 2,3,7,8-tetrachlorodibenzo-*p*-dioxin has been shown to disrupt a range of maturational processes, depending upon the specific timing of exposure (Kerkvliet 2002). However, to validate findings from laboratory models of immune dysfunction, assessment of developmental immunotoxicity in epidemiologic studies is needed.

Several studies have reported associations between developmental PCB exposures and a variety of immune function parameters (Belles-Isles et al. 2002; Bilrha et al. 2003; Levin et al. 2005), with some studies focusing on immunoglobulin concentrations at different ages (Dewailly et al. 2000; Nagayama et al. 1998; Weisglas-Kuperus et al. 2000). Among immune system properties that are feasible and appropriate for population studies, vaccination responses are of substantial public health relevance (van Loveren et al. 1999). The serum concentrations of antibodies against vaccine toxoids constitute a promising tool for studies of children, who have been primed by standardized doses of foreign antigens at well-defined ages as part of routine immunization programs. Up-regulation of the T-helper cell type 1 functional capacities crucial for vaccine antibody responses against T-cell–dependent antigens occurs during early postnatal development. Accordingly, children may be particularly vulnerable to immunotoxicity caused by exposures to environmental chemicals during early postnatal development (Dietert 2008). In two birth cohorts from the Faroe Islands, we recently found that the antibody response to diphtheria toxoid was significantly decreased at 18 months of age and, in an older cohort, that the tetanus toxoid antibody response was affected mainly at 7 years of age. The early postnatal PCB exposure level was the most important predictor of a decreased vaccination response (Heilmann et al. 2006).

The Faroe Islands provide a unique opportunity to study PCB immunotoxicity. Exposures vary widely because of the PCB contamination of pilot whale blubber, a traditional food item favored by many Faroese, but not all (Grandjean et al. 2001). To further explore the possible effect of PCBs on vaccine response, we have now completed follow-up at 7 years of age of the younger of the two cohorts previously examined (Heilmann et al. 2006), and invitations were extended to all cohort members, thereby securing a study group five times larger than the one originally studied.

## Methods

### Birth cohort and clinical examinations

The Faroe Islands constitute a Nordic fishing community located in the North Atlantic between Shetland and Iceland. A birth cohort was formed from consecutive births during 1999–2001. Informed consent was obtained from a total of 656 mothers in connection with consecutive spontaneous singleton births at term.

In the Faroes, the government offers free medical care, and childhood vaccinations are carried out according to a schedule similar to the one used in Denmark. The first childhood vaccination, at age 3 months, includes immunization against diphtheria and tetanus, along with pertussis, polio, and *Haemophilus influenzae* type b. Repeat inoculations are given at ages 5 and 12 months, and a booster vaccination against diphtheria and tetanus is given at age 5 years.

To examine possible changes in antibody responses to vaccinations, the birth cohort was prospectively followed until age 7 years. A subgroup of these cohort members first came for a follow-up study at 12 and 18 months of age (Heilmann et al. 2006), and 116 children had sufficient serum for analysis of PCBs. Most of these children also participated in the subsequent follow-up. The next examination occurred at approximately 5 years of age, before the child had received the booster vaccination; 532 cohort members participated and successfully provided a serum sample for the analyses (81%). A follow-up examination was then scheduled for the same children about 1 month after the booster, as is the routine procedure in vaccination immunogenicity studies. A serum sample was obtained from a total of 456 children. The birth cohort members were again invited when they were about 7.5 years of age. Of 464 cohort members (71%) examined, 43 had not participated 2 years before; 110 subjects examined at age 5 years did not participate at age 7.

The study protocol was approved by the ethical review committee serving the Faroe Islands and by the institutional review board at Harvard School of Public Health.

### Exposure assessment

Exposures to marine contaminants were assessed from analysis of biological samples first obtained from the mother at the last antenatal examination at week 32 of pregnancy, followed by transitional milk samples at postparturition days 4–5, and serum samples from the child at successive clinical examinations. Serum analyses were conducted by gas chromatography with electron capture detection at the University of Southern Denmark. As before (Heilmann et al. 2006), the accuracy and reliability of the data were ensured by including quality control serum samples [excess serum samples from the German External Quality Assessment Scheme (G-EQUAS) round-robin program as well as spiked serum pools] in each analytical batch of samples, calibration standards, and reagent and serum blanks. Excellent results were obtained in intercalibration between these laboratories and in the G-EQUAS organized by the German Society of Occupational Medicine. The milk analyses were performed by similar methodology by the Department of Environmental Health, State Agency for Health and Occupational Safety of Schleswig-Holstein, Germany (Schade and Heinzow 1998). This laboratory serves as a reference laboratory for analyses of environmental chemicals in milk. To avoid problems with congeners not assessed and concentrations below the detection limit, a simplified ∑PCB concentration was calculated as the sum of congeners CB-138, CB-153, and CB-180 multiplied by 2 (Grandjean et al. 1995). The ∑PCB concentration was expressed in relation to the total lipid concentration determined using the Cypress Diagnostics kit (Langdorp, Belgium). In addition to the ∑PCB concentration, the weighted sum of the three main mono-*ortho*–substituted congeners CB-105, CB-118, and CB-156 was calculated using toxicity equivalency factors to obtain the dioxin equivalent concentration. The analyses also provided the concentrations of the pesticide metabolite *p,p*-dichlorodiphenyldichloroethene (*p,p*-DDE), hexachlorobenzene, and β-hexachlorocyclohexane, which occurred at lower levels but were detectable in almost all samples. However, because of close correlations with ∑PCB, these additional analytes were not examined further. As a measure of methylmercury exposure, total mercury concentrations were measured in cord blood, maternal hair at parturition, and hair and blood from the children at the clinical examinations (Grandjean et al. 2010).

### Vaccines and antibody measurements

All infants were vaccinated according to the official Danish/Faroese vaccination program. Tetanus toxoid and diphtheria toxoid are both classical protein antigens that depend on T-helper cells for both primary and recall antibody responses. Toxoids for the routine childhood vaccinations and boosters were from Statens Serum Institut (SSI, Copenhagen, Denmark). All children received the same amount of diphtheria and tetanus toxoid and associated alum adjuvant. However, because of changes in the formulation from 1 September 2003, the diphtheria-tetanus vaccine also contained pertussis antigen and (from 1 July 2004) polio; both had previously been given by injection separately from diphtheria-tetanus. None of the vaccines contained mercury-based preservatives.

Serum concentrations of antibodies against tetanus toxoids were measured by SSI using enzyme-linked immunosorbent assay (Hendriksen et al. 1988), whereas antibodies against diphtheria toxoids were measured using a standard Vero cell–based neutralization assay employing 2-fold dilutions of serum samples (Miyamura et al. 1974). For both assays, calibration was performed using international and local standard antitoxins.

### Data analysis

Antibody concentrations were first treated as continuous variables, because logarithmic transformations reasonably approached a Gaussian distribution. Associations of PCB exposure with antibody concentrations were determined using standard regression techniques. Antibody concentrations were log transformed to obtain normally distributed residuals with a homogeneous variance. As obligatory covariates, we included sex and age only. Both antibody outcomes obtained after the 5-year booster were adjusted for the booster status (i.e., with and without other vaccines), and the analysis of the immediate post-booster response also included the time interval since booster inoculation. Because of the clinical importance of protective antibody concentrations > 0.1 IU/mL, odds ratios (ORs) were calculated for the effect of PCB exposure on the probability of having an antibody concentration below this limit.

PCB exposure parameters were entered into the regression models, one at a time, after logarithmic transformation. Because of the transformations, measures of effects could be expressed as the relative change (percent) of the outcome variables for each doubling of the exposure. Residual plots were used to assess the model fit, and the possible significance of second- and third-order terms was determined. Additional covariates—birth weight, maternal smoking during pregnancy, and duration of breast-feeding—were then included to determine the possible influence on the PCB regression coefficients. Separate regressions were also made to estimate the possible impact of methylmercury exposure.

The large number of missing values for the PCB concentration at 18 months complicated the estimation of a possible negative association with this exposure. To remedy the problem, we used the multiple imputation method (Rubin 1987) in SAS version 9.1 (SAS Institute Inc., Cary, NC, USA). Thus, for children without this exposure measure, the PCB concentration was imputed based on the known association with the observed PCB concentrations at birth and at 5 years, as well as the length of the breast-feeding period. Using this approach, the relatively large number of children who missed information on the 18-months concentration could be included in the regression model. Compared with the standard complete case analysis, the imputation would be expected to lead to a decrease in standard errors of regression coefficients. All two-tailed *p*-values < 0.05 were considered statistically significant.

After the booster vaccination at age 5 years, cohort members were examined at different intervals. A nonlinear model for the time-dependent change was therefore generated using a cubic spline function, which was used to adjust the serum antibody concentrations before the regression analyses.

## Results

Major characteristics of the 587 cohort members contributing serum samples at ages 5 and/or 7 years are included in Table 1. The pre-booster concentration of both diphtheria and tetanus antibodies correlated well with the results at the two subsequent examinations (Table 2). Similarly, correlations between log-transformed tetanus and diphtheria concentrations obtained at the same examination were 0.45, 0.32, and 0.43 at the three occasions (before booster, 1 month after booster, and at 7 years, respectively). Although boys had higher antibody concentrations than girls and also had higher PCB exposures at ages 5 and 7 years (results not shown), the extended set of covariates appeared to cause little potential for confounding.

Results of multiple regression analyses with adjustment for sex and age indicated a clear inverse association between the pre-booster diphtheria-specific antibody concentration and PCB concentrations in maternal milk and in the child’s serum at 18 months of age, although the latter comparison was based on a much smaller number of subjects than the other comparisons. PCB levels measured at age 5 years were not associated with the pre-booster tetanus response at this age (Table 3). Further adjustment for additional covariates did not materially affect these results.

**Figure 1:**
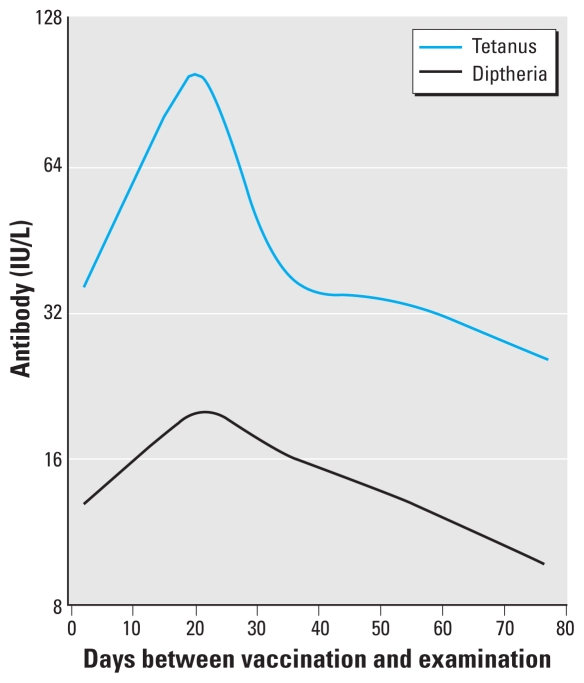
Time-dependent change in serum antibody concentrations against tetanus and diphtheria in 455 Faroese children after booster vaccination at age 5 years, as modeled by a cubic spline function adjusted for sex, age, and booster type. Results are shown for girls at age 5.2 years inoculated with a booster without additional vaccines.

Serum antibody concentrations after the 5-year booster vaccination varied according to the time interval between the booster vaccination and serum collection (Figure 1). Five children had a negative interval (i.e., they were examined before the booster vaccination) and were therefore excluded from the analysis of post-booster antibody concentrations. A cubic spline function was used for adjustment of the data used in these regression analyses. Each of the two newer booster vaccinations that included additional vaccines were associated with lower antibody responses for both tetanus and diphtheria (*p* < 0.05); adjustment for the booster type was therefore also included in these models as well as for the 7-year data.

Although none of the PCB concentrations at different time points reached statistical significance as predictors at the post-booster examination, all showed an inverse association with the antibody concentrations. Thus, doubling of PCB concentrations in maternal serum [−7.5%; 95% confidence interval (CI), −18.8 to 5.4; *p* = 0.24], milk (−8.2%; 95% CI, −16.9 to 1.5; *p* = 0.10) and 18-month serum (−8.3%; 95% CI, −26.4 to 14.2; *p* = 0.43) suggested similar relative declines in the diphtheria antibody concentration. Although inclusion of the additional covariates did not materially affect these results, the concurrent 5-year serum PCB concentration became a significant predictor (−11.6%; 95% CI, −19.4 to −3.0; *p* = 0.01) for the diphtheria antibody concentration after adjustment for all covariates.

At age 7 years, six children were excluded because they had received an additional booster at some point after the routine booster at 5 years of age. Children inoculated with the two combination vaccines containing pertussis and/or polio showed a lower antibody response toward tetanus and diphtheria, and combination booster was therefore included as a covariate along with sex and age. Again, we saw inverse associations with PCB exposure, although not as strong as at age 5 years (Table 4). Only the serum PCB concentration at age 18 months approached statistical significance with regard to the decrease in diphtheria antibody concentrations, although the response was almost as strong with regard to tetanus.

Because of the pronounced association with the 18-months serum PCB concentration, which was obtained only for about one-fifth of the cohort, we conducted further analyses using the imputed data. These results strengthened the observations, and an inverse association was now apparent for tetanus at age 7 years (Table 5).

We further identified the children who had antibody concentrations below a clinically protective level of 0.1 IU/mL. As anticipated, the highest number was at the pre-booster examination at age 5 years, with 202 (37%) children having a low diphtheria concentration and 141 (26%) having a low tetanus concentration. The numbers of children with inadequate antibody concentrations were much less (7% and 4%, respectively) at age 7 years, 2 years after booster vaccination. The ORs for inadequate antibody concentrations associated with a doubling of PCB concentrations are shown in Table 6. In general, children showing insufficient protection against diphtheria had experienced higher PCB exposures, as reflected by the concentrations in milk and in serum concentrations at ages 18 months or 5 years. Among the limited number of children with available exposure data from age 18 months, the only two children with insufficient protection against diphtheria at age 7 years had PCB concentrations of 3.9 μg/g lipid and 5.3 μg/g lipid—among the five highest in the group, a statistically significant deviation (*p* = 0.04) from an expectation of no association.

Mercury exposure parameters showed variable associations with the antibody concentrations without a clear pattern. The strongest negative association was for the hair-mercury concentration at age 5 years with regard to the diphtheria antibody concentration at age 7 years (−16.3; 95% CI, −31.3 to 1.9; *p* = 0.08), but this association was substantially attenuated when adjusted for PCB (−5.6; 95% CI, −23.9 to 17.2; *p* = 0.60). Adjustment for mercury had only a negligible effect on the regression coefficients for PCB (data not shown).

## Discussion

Our study provides support for the notion that PCB immunotoxicity has clinical implications in humans. A major strength is that a population-based birth cohort was followed for > 7 years, and samples for exposure assessment and vaccine antibody assessments were collected at several time points. The results showed that the estimated effect on antibody concentrations depends on the time window that the PCB analysis represents. For example, the maternal pregnancy serum concentration, which reflects intrauterine PCB exposure, showed only a small and not statistically significant association with antibody concentrations. The same was true for serum PCB concentrations determined at the same time as antibody assessments, at ages 5 and 7 years, that is, in cross-sectional analyses. However, the serum PCB at age 18 months tended to show the strongest association, thus suggesting that body burdens at this age may coincide with a highly vulnerable stage of immune system development. The average PCB concentration at 18 months was slightly higher than the average maternal level during pregnancy (Table 1), probably because the former represents the child’s total accumulation of PCBs both from intrauterine exposure and from lactational transfer during the full period of breast-feeding. In the Faroes, extended breast-feeding is common, with average exclusive and total duration of 4.6 months and 9.8 months in this birth cohort (Grandjean et al. 2010). Thus, the transfer of PCBs and other potential immunotoxicants via human milk (Grandjean et al. 1995) is of particular concern because of the vulnerability of the immune system during the perinatal period.

The findings of this study are in agreement with our previous findings from two smaller groups of Faroese children, one of which constituted a subgroup of the cohort included in the present study (Heilmann et al. 2006). At the 18-month examination of a subgroup of the cohort presented in this report, the diphtheria-specific antibody showed a clear decrease associated with increases in the concomitant serum PCB concentration. On the other hand, our previous study (Heilmann et al. 2006) showed an effect of perinatal PCB exposure on the tetanus antibody concentration at 7 years of age, although these children had undergone a different schedule of vaccinations. Although the amounts of tetanus and diphtheria toxoids were the same in all booster vaccines applied in the studies, polio and pertussis antigens were added to the vaccines during the duration of this study. The observed antibody responses to diphtheria and tetanus toxoids in the two studies may therefore not be fully comparable. Still, in our previous study, a doubling of the PCB exposure at 18 months was associated with a decrease in the concomitant antibody response to the diphtheria toxoid by 24%, and the decrease in the tetanus toxoid antibody at age 7 years was 16% for each doubling of the prenatal exposure level (Heilmann et al. 2006). These responses are similar to the largest effects of about 20% observed in this study (Table 5).

The results were clearly affected by the time of the examination with regard to the antigen stimulation. The antibody concentrations obtained immediately after the booster showed a high degree of variability associated with the time interval before serum collection (Figure 1). Adjustment for the time was attempted by including a spline term for the time interval since vaccination, but only the average time-dependent change could be modeled, and the result for each child therefore remained somewhat imprecise. Although a routine in vaccinology (Siegrist 2008), these findings suggest that collection of a sample about 1 month after vaccination is not ideal in studies of immunotoxicity. Although the average peak response occurred after 20 days for both tetanus and diphtheria, adverse effects on antibody concentration may be masked by individual differences in the time-dependent change in serum antibody concentrations. However, it is noteworthy that, in this case, the concurrent PCB concentration showed the strongest association with a decreased post-booster antibody response.

Our results emphasize that PCB exposures should be determined at an age that reflects the most vulnerable window of development. Although several PCB congeners are persistent and have elimination half-lives of several years, serum concentrations in children change because of the dilution resulting from an expanded lipid compartment (Grandjean et al. 2008). Age 18 months appears to represent an approximate peak age with regard to immune system vulnerability, and the present data therefore suggest that representative serum samples should be secured between ages 1 and 2 years for future studies of human immunotoxicity.

Among possible weaknesses of this study, serum collection that was incomplete at age 18 months appeared to be of the greatest relevance to the findings. However, partial adjustment for the missing data was achieved through statistical modeling involving multiple imputations. This method relies on the linear association between log-transformed PCB concentrations at different ages and assumes that PCB values are missing at random. Although some deviation from this assumption is possible, the multiple imputation method is less likely to be biased than the standard complete case analysis (Rubin 1987). Although several potential covariates were examined and included in multiple regression analyses, few of them played any role at all. As previously reported (Heilmann et al. 2006), most of the variability in serum antibody concentrations is unexplained, and compared with the covariates, PCB exposure was a remarkably strong predictor. The Faroese population is also exposed to contaminants other than PCBs, such as *p,p*-DDE, hexachlorobenzene, and β-hexachlorocyclohexane. Because they correlate with PCB, this study could not assess whether they played any independent role. However, they occurred in lower concentrations than the PCBs, and available data suggest that any immunotoxicity is likely to be relatively weak (Barnett et al. 1987; Das et al. 1990). Methylmercury exposure did not seem to affect the outcomes.

The implications of the results need to take into account that antibody responses of normal children to routine prophylactic vaccinations can vary substantially, but the reasons for this wide variation are poorly understood. An antibody concentration > 0.1 IU/mL is considered essential to achieve full long-term protection in accordance with the public health purpose of routine vaccinations. The clinical importance of our findings is, therefore, that the PCB exposure may increase the risk of a child not being protected against diphtheria and possibly tetanus, despite a full schedule of vaccinations.

These results should also be considered in light of the range of immune processes involved in antibody formation, including antigen presentation, T-lymphocyte function, and B-lymphocyte function (Dietert 2008; van Loveren et al. 1999). Because of the involvement of several key components of the immune system, antibody concentrations triggered by standardized antigen stimulations may reflect the overall efficacy of the immune system in relation to infection. The PCB-associated decreases in antibody concentrations may therefore also relate to potential adverse consequences of immune system deficits beyond the protection against the two specific bacteria.

## Figures and Tables

**Table 1:** Characteristics, with arithmetic mean ± SD and geometric mean (interquartile range) values, of 587 birth cohort members examined for serum concentrations of specific vaccine antibodies at ages 5 and 7 years.

Variable	*n*	Result
Maternal age at parturition (years)	587	29.3 ± 5.1
Previous births (none/one/at least two) (%)	587	27.7/32.8/39.5
Smoking during pregnancy (no/yes) (%)	587	71.4/28.6
Gestational age (weeks)	587	39.6 ± 1.3
Birth weight (g)	587	3,721 ± 509
Sex (boys/girls) (%)	587	53.1/46.9
Maternal serum ∑PCB concentration (μg/g lipid)	427	1.25 (0.83–1.90)
Milk ∑PCB concentration (μg/g lipid)	521	1.34 (0.85–2.13)
Duration of exclusive breast-feeding (months)	565	4.6 ± 2.0
Total duration of breast-feeding (months)	569	9.8 ± 6.6
Serum ∑PCB concentration at age 18 months (μg/g lipid)	114	1.17 (0.65–2.11)
Serum ∑PCB concentration at age 5 years (μg/g lipid)	532	1.14 (0.70–1.93)
Serum ∑PCB concentration at age 7 years (μg/g lipid)	487	0.75 (0.43–1.38)
Age at pre-booster 5-year examination (years)	545	4.95 ± 0.06
Age at post-booster examination (years)	461	5.20 ± 0.12
Age at 7-year examination (years)	565	7.53 ± 0.11

**Table 2:** Geometric mean (and interquartile range) serum antibody concentrations (IU/L) in 587 children examined on up to three occasions at ages 5–7 years.

	*n*	Geometric mean (interquartile range)	Correlation with pre-booster
Tetanus			
5 years, pre-booster	529	0.22 (0.10–0.52)	(1)
5 years, post-booster	455	35 (16–96)	0.53
7 years	464	1.59 (0.65–4.6)	0.62

Diphtheria			
5 years, pre-booster	529	0.12 (0.05–0.40)	(1)
5 years, post-booster	455	13 (6.4–26)	0.49
7 years	464	0.68 (0.40–1.60)	0.51

**Table 3:** Relative change in serum antibody concentrations at age 5 years (before booster vaccination) associated with a doubling in developmental exposure to PCBs, as indicated by the concentrations in biological samples obtained at different times.

	*n*	Change (%)	95% CI	*p*-Value
Tetanus				
Pregnancy serum PCB	390	−0.67	−14.4 to 15.3	0.93
Milk PCB	477	−0.89	−11.6 to 11.1	0.88
Child serum PCB, 18 months	107	−8.3	−26.1 to 13.7	0.42
Child serum PCB, 5 years	532	0.6	−8.7 to 10.8	0.91

Diphtheria				
Pregnancy serum PCB	390	−12.9	−26.0 to 2.6	0.10
Milk PCB	478	−13.9	−24.2 to −2.4	0.02
Child serum PCB, 18 months	106	−29.3	−45.5 to −8.3	0.01
Child serum PCB, 5 years	533	−6.4	−15.9 to 4.2	0.23

Results are from regression model with adjustment for age and sex.

**Table 4:** Relative change in serum antibody concentrations at age 7 years associated with a doubling in developmental exposure to PCBs, as indicated by their concentrations in biological samples obtained at different times from members of a Faroese birth cohort.

	*n*	Change (%)	95% CI	*p*-Value
Tetanus				
Pregnancy serum PCB	329	8.0	−8.9 to 28.0	0.37
Milk PCB	402	−3.6	−15.4 to 9.9	0.58
Child serum PCB, 18 months	67	−11.6	−32.8 to 16.3	0.37
Child serum PCB, 5 years	410	−4.5	−15.0 to 7.3	0.42
Child serum PCB, 7 years	438	−1.3	−11.0 to 9.4	0.80

Diphtheria				
Pregnancy serum PCB	329	1.3	−13.3 to 18.5	0.87
Milk PCB	402	−2.0	−13.6 to 11.0	0.75
Child serum PCB, 18 months	67	−17.0	−33.1 to 3.0	0.09
Child serum PCB, 5 years	410	−1.5	−11.7 to 9.8	0.78
Child serum PCB, 7 years	438	1.4	−7.8 to 11.5	0.78

Results are from regression model with adjustment for age, sex, and booster status.

**Table 5:** Effect on antibody concentrations at ages 5 years (pre-booster) and 7 years associated with PCB exposures reflected by the serum concentration at 18 months after multiple imputation to allow for missing observations among the 587 members of a Faroese birth cohort.

Antibody measure	Change (%)	95% CI	*p*-Value
Tetanus			
5 years	−6.0	−16.5 to 5.7	0.30
7 years	−21.7	−33.1 to −8.2	0.003

Diphtheria			
5 years	−15.4	−25.8 to −3.5	0.01
7 years	−18.3	−31.9 to −2.1	0.03

Results are from regression model with adjustment for age and sex; results at 7 years were also adjusted for booster status.

**Table 6:** ORs (95% CIs) and *p*-values describing the effect of a doubling exposure concentration on the risk of an antibody concentration below a clinically protective level of 0.1 IU/mL in Faroese birth cohort members examined at ages 5 and 7 years (*n* = 587).

	Antidiphtheria toxoid				Antitetanus toxoid			
	5 years		7 years		5 years		7 years	
PCB exposure	OR (95% CI)	*p*-Value	OR (95% CI)	*p*-Value	OR (95% CI)	*p*-Value	OR (95% CI)	*p*-Value
Pregnancy	1.15 (0.91–1.45)	0.24	0.97 (0.62–1.52)	0.89	1.19 (0.93–1.53)	0.17	0.93 (0.53–1.62)	0.79
Milk	1.34 (1.11–1.61)	0.002	1.03 (0.71–1.50)	0.88	1.11 (0.91–1.36)	0.30	1.06 (0.63–1.79)	0.82
18 months^a^	1.30 (1.08–1.57)	0.006	1.58 (0.88–2.83)	0.12	1.15 (0.92–1.44)	0.22	1.64 (0.96–2.81)	0.07
5 years	1.19 (1.01–1.39)	0.03	1.09 (0.77–1.53)	0.64	1.09 (0.92–1.29)	0.33	1.12 (0.72–1.75)	0.61
7 years	—	—	1.01 (0.74–1.39)	0.93	—	—	1.12 (0.71–1.75)	0.63

Results are from logistic regression model with adjustment for age and sex; results at 7 years were also adjusted for booster status.

a Based on multiple imputation data.
